# Successful anti-IL-6 treatment for interstitial lung disease associated with STAT3 gain-of-function: a case report and literature review

**DOI:** 10.3389/fped.2025.1577746

**Published:** 2025-07-09

**Authors:** Peng Zhou, Meifang Zhu, Yuting Pan, Jing Jin, Zhidan Fan, Haiguo Yu

**Affiliations:** Department of Rheumatology and Immunology, Children’s Hospital of Nanjing Medical University, Nanjing, China

**Keywords:** STAT3, gain-of-function, inborn error of immunity, interstitial lung disease, tocilizumab

## Abstract

Autosomal dominant gain-of-function (GOF) variants in the signal transducer and activator of transcription 3 (*STAT3*) result in an inborn error of immunity characterized by multi-organ autoimmunity and lymphoproliferation. In this study, we retrospectively analyzed a rare case of *STAT3* GOF mutation with thrombocytopenia, immunoglobulin deficiency, and recurrent respiratory infections. Whole-exome sequencing revealed a heterozygous mutation (c. 2144C > T, p. P715l) in the *STAT3* gene. The patient initially received only anti-infective and immunoglobulin-supportive therapies at an external hospital, which proved unsatisfactory. Over time, the patient developed severe interstitial lung disease (ILD) and arthritis, which were effectively managed with tocilizumab at our hospital. This case underscores the importance of early diagnosis and timely initiation of biological therapy for the management of ILD with *STAT3* GOF mutations.

## 1 Introduction

Signal transducer and activator of transcription 3 (STAT3) belongs to the STAT protein family, which includes seven members and is widely expressed in various immune-related cells. STAT3 plays a central role in regulating cell survival, proliferation, differentiation, and effector functions (1). Human STAT3 is a 770-amino acid protein comprising six structural domains: the N-terminal domain, coiled-coil domain, DNA binding domain, linker domain, Src homology-2 domain, and transcriptional activation (TAD) domain in the C-terminal. Notably, the TAD domain plays a role in regulating phosphorylation (2). A gain-of-function (GOF) mutation in *STAT3* results in an inborn error of immunity (IEI), characterized by multi-organ autoimmunity and lymphoproliferation (3). In this study, we describe a case of IEI with a predominant interstitial lung disease (ILD) phenotype and arthritis due to *STAT3* GOF mutation. The manifestations of ILD and arthritis in the patient were effectively managed with tocilizumab.

## 2 Case description

**Figure 1:**
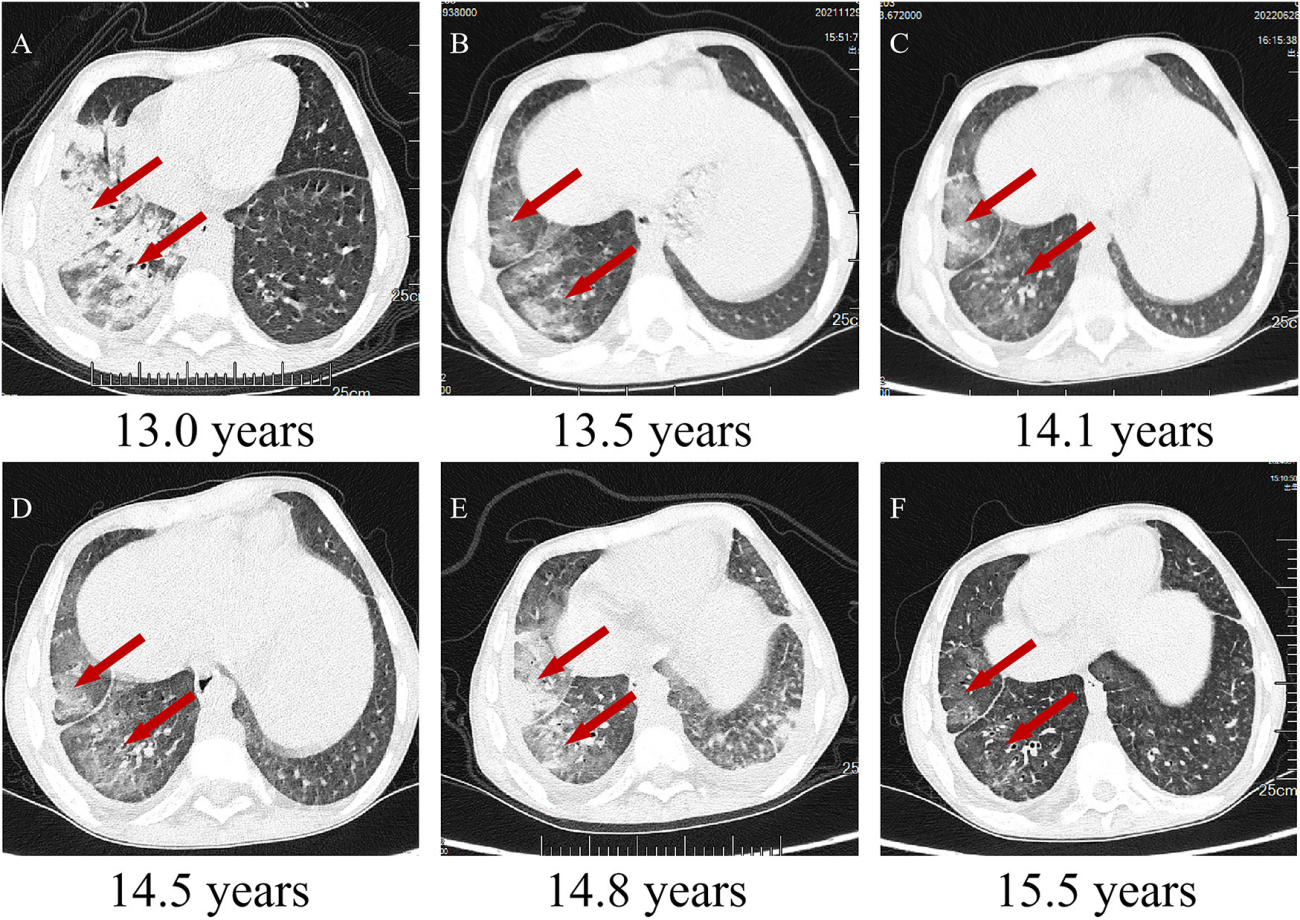
Chest CTs of the patient. **(A–F)** Chest CTs following regular routine treatment with tocilizumab. Inflammation of the middle and lower lobes of the right lung with ground-glass opacities, reticulation, airspace consolidation, and traction bronchiectasis (indicated by red arrows).

A 12-year-old boy with a 3-month history of pain in the left knee, left elbow, and right ankle presented to our clinic. In addition to arthralgia, the patient also experienced chronic cough for one month with viscous sputum and dyspnea particularly noticeable at night or after exercise. Digital clubbing was also observed. The patient presented with markedly elevated serum Krebs von den Lungen-6 (KL-6) levels (2,887 U/ml; normal <500 U/ml), a biomarker for disease severity assessment in ILD. High-resolution computed tomography (HRCT) revealed characteristic interstitial lung abnormalities including ground-glass opacities, reticulation, airspace consolidation, and traction bronchiectasis (Figure 1A). Pulmonary function testing demonstrated impaired gas exchange with diffusing capacity for carbon monoxide (DLCO) at 70% of predicted value. Functional capacity was compromised as evidenced by 6 min walk distance <80% predicted and oxygen desaturation during exertion (SpO₂ 95% at rest vs. 84% with exercise). Bronchoscopy with bronchoalveolar lavage (BAL) showed lymphocytic-predominant inflammation (lymphocytes 60%, neutrophils 15%, monocytes 25%), while metagenomic next-generation sequencing (mNGS) returned negative for pathogens. These collective findings confirmed associated interstitial lung disease. Other laboratory investigations after admission revealed anemia, thrombocytopenia, and hypogammaglobulinemia (Table 1). Magnetic resonance imaging of the left knee joint revealed periarticular inflammatory lesions accompanied by soft tissue swelling, consistent with the imaging characteristics of arthritis. Furthermore, abdominal ultrasonography demonstrated hepatosplenomegaly.

**Table 1:** Immune laboratory values for the patient.

Parameter	Measurements of the patient	Reference range
C-reactive protein	20.7 mg/L	0–10 mg/L
White blood cells	6.1 × 10^9^/L	4.6–11.3 × 10^9^/L
Hemoglobin	104 g/L	131–179 g/L
Platelets	123 × 10^9^/L	148–399 × 10^9^/L
Neutrophils	2.91 × 10^9^/L	1.9–7.9 × 10^9^/L
Lymphocytes	2.56 × 10^9^/L	1.5–4.2 × 10^9^/L
CD3^+^	1,548.45 cell/ul	950–1,960 cell/ul
CD3^+^ CD8^+^	1,086.75 cell/ul	315–803 cell/ul
CD3^+^ CD4^+^	272.46 cell/ul	446–1,100 cell/ul
IgG	<1.53 g/L	8.27–14.17 g/L
IgA	<0.265 g/L	0.86–1.92 g/L
IgM	<0.181 g/L	1.22–2.56 g/L
IL-6	8.31 pg/ml	≤5.4 pg/ml

The medical history of the patient indicated recurrent respiratory infections starting at the age of 4 years, for which the patient received antibiotic therapy at external hospitals. At 8 years of age, thrombocytopenia and hypogammaglobulinemia were first documented, with persistently subnormal levels confirmed through repeated testing over the next 8 months. Consequently, regular intravenous immunoglobulin (IVIG) replacement therapy was initiated every 3 months (Supplementary Figure S1). The proband has a healthy younger sister. The parents were non-consanguineous and had no family history of inherited genetic disorders.

## 3 Diagnostic assessment

**Figure 2:**
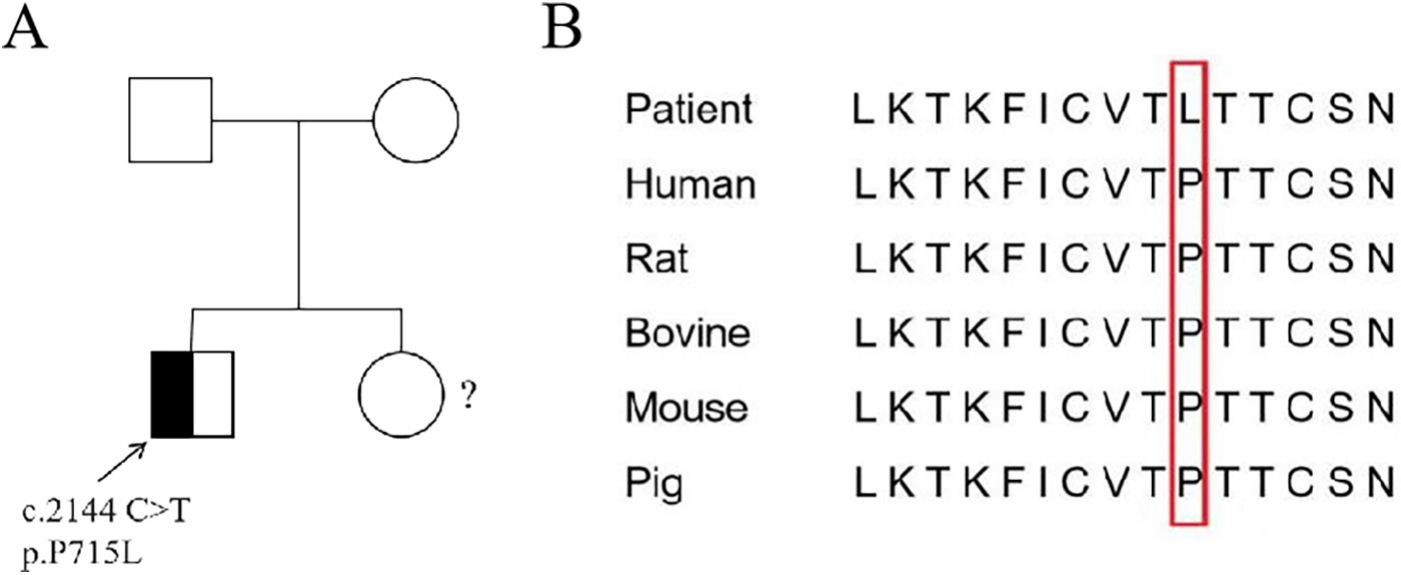
Sanger sequencing and conservative analysis of *STAT3* in the patient. **(A)** The patient exhibited a heterozygous mutation c.2144C > T in exon 11 of the *STAT3* gene (the arrow indicates the mutation location). The parents of the patient did not have this mutation and are wild type.?: The sister did not undergo WES. **(B)** Conservative comparison of STAT3 across five species showed that P 715 is highly conserved.

The patient underwent whole-exome sequencing (WES) at an external hospital to investigate the etiology of thrombocytopenia and hypogammaglobulinemia at the age of 8 years. His younger sister did not undergo WES due to parental refusal (Figure 2A). WES revealed a heterozygous mutation in exon 22 of the *STAT3* gene, resulting in a missense mutation that substitutes proline with leucine at amino acid 715 of the encoded protein (c.2144C > T, p.P715l). Family segregation analysis revealed that neither parent harbored a mutation at this locus (Figure 2A). Conservative analysis revealed that the mutation site is highly conserved among species (Figure 2B). At the time, this variant was not documented in established databases or published literature, resulting in an indefinite diagnosis and the initiation of IVIG therapy at the external hospital. According to GenIA, the variant was initially reported in one boy and one woman from the Czech Republic. In 2020, Mauracher et al. (4) described a female patient carrying the same variant who presented with autoimmune hemolytic anemia and erythroid aplasia. *in vitro* functional charcterization conducted by the authors showed increased STAT3 phosphorylation by western blot and increased transactivation activity, using a luciferase reporter assay, in both resting and stimulated (Interleukin-6) conditions in HEK cells (4). These experimental findings collectively demonstrate the pathogenicity of this variant.

### 3.1 Therapeutic intervention

The patient was treated with monthly intravenous tocilizumab (8 mg/kg) along with quarterly IVIG administration for immunomodulatory support and infection prevention. Mild thrombocytopenia and anemia were managed conservatively with regular monitoring.

### 3.2 Follow-up and outcomes

**Figure 3:**
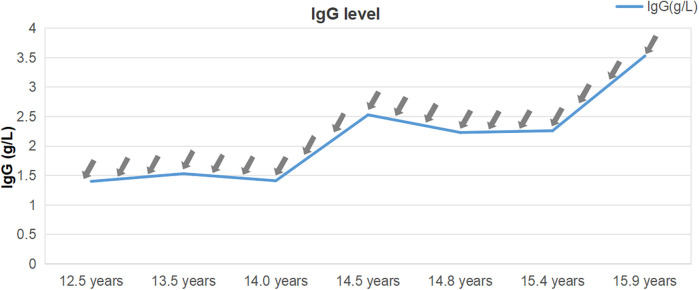
Immunoglobulin G levels from diagnosis to present. The immunoglobulin levels remained 1–4 g/L for a long period, notably below the reference range, despite receiving immunoglobulin G injections (0.5 mg/kg) every 2–3 months (indicated by grey arrows).

The patient showed a marked improvement in arthritic symptoms following treatment with tocilizumab, and the ILD symptoms were also effectively managed. The patient maintained normal oxygen saturation levels, demonstrated gradual resolution of exertional dyspnea, and exhibited complete regression of digital clubbing. Following therapeutic intervention, significant improvement was observed: KL-6 decreased substantially to 390 U/ml. Follow-up HRCT demonstrated regression of ground-glass opacities, reticulation, and bronchial dilation (Figure 1B). DLCO increased to 85% predicted, 6-minute walk distance achieved 90% predicted and Oxygen saturation normalized (99% at rest, 93% on exertion). Following tocilizumab and IVIG therapy, the patient demonstrated partial recovery of immunoglobulin level (Figure 3), although they remained below the normal range (8.27–14.17 g/L). A marked reduction in infection frequency supported the continuation of the original IVIG dosing regimen for sustained supportive care. However, during longitudinal follow-up, the patient exhibited persistent failure to thrive (FTT), with anthropometric parameters revealing progressive deterioration. Specifically, at 14 years of age, height was 139 cm (z-score: −3.51) and weight was 27 kg (z-score: −2.67), and at 15 years of age, height was 140 cm (z-score: −4.07) and weight remained at 27 kg (z-score: −3.15). Parental heights were 174 cm (father) and 162 cm (mother), yielding a calculated mid-parental target height of 174.5 cm.

## 4 Literature review of patients with *STAT3* GOF and ILD

Studies on *STAT3* GOF mutation-associated ILD were comprehensively searched on PubMed up to December 31, 2024, using the search terms “*STAT3*,” “gain-of-function,” and “ILD.” We identified seven cases of *STAT3* GOF-associated ILD treated using tocilizumab. Subsequently, we collected and analyzed the basic information, symptoms, treatments, and prognosis of these seven patients, including three males and four females, aged 2–16 years (Table 2). All patients exhibited three or more clinical phenotypes. Two of them were diagnosed with lymphocytic interstitial pneumonitis (LIP) on pathological examination (P3, P6). The earliest reported onset of ILD among them was at 3 months of age (5). This patient exhibited rapid ILD progression, culminating in acute respiratory distress syndrome by 12 months of age. Combination therapy with tocilizumab and ruxolitinib, augmented by extracorporeal membrane oxygenation support, significantly improved clinical and radiographic outcomes in ILD. One patient developed ILD at 15 years of age and declined targeted drug therapy (P7). Ultimately, the patient succumbed to progressive worsening of ILD and heart failure (6). The remaining six patients were treated with tocilizumab or a combination of JAK inhibitors. One patient was treated with a JAK inhibitor following the failure of tocilizumab but died of recurrent infections and pneumonia (P6). Among the remaining five patients, two were treated with tocilizumab alone, whereas the other three received combination therapy.

**Table 2:** Clinical manifestations and IL-6-directed therapies in patients with *STAT3* GOF and ILD.

Patient number	Patient 1 (present case)	Patient 2 (4)	Patient 3 (22)	Patient 4 (9)	Patient 5 (11)	Patient 6 (12)	Patient 7 (6)
Patient Age/Sex	12 year/M	7 year/F	10 year/F	2 year/F	9 year/F	15 year/M	16 year/M
Mutation and protein change	c.2144C > T p.P715l	c.454C > T p.R152W	c.1032G > C p.Q344H	c.1973A > T p.Lys658Met	E415l	c.2144C > T p.P715l	c.508G > C p.D170H
Pulmonary disease	ILD	ILD	LIP with diffuse ground glass pulmonary opacities	ILD	ILD	LIP with restrictive disease and severe diffusion defect	ILD
Infection	Yes	Yes	No	No	No	No	Yes
Lymphoproliferation	SPM	Diffuse LAD and SPM	Diffuse LAD and SPM	Diffuse LAD and SPM	No	Diffuse LAD and SPM	SPM
Cytopenia	Thrombocytopenia anemia	Neutropenia thrombocytopenia	No	Thrombocytopenia anemia	No	Thrombocytopenia	Autoimmune cytopenia
Hepatopathy	HTM and widening of hepatic portal vein diameter	HTM	Hepatic dysfunction	No	No	Nodular regenerative hyperplasia	HTM
Enteropathy	No	No	Yes	Yes	Yes	Yes	Yes
Growth	FTT	FTT	FTT	FTT	FTT	FTT	No
Endocrinopathies	No	No	No	Type 1 diabetes	Diabetes	Hashimoto’s thyroiditis	Diabetes Thyroid disease
Arthritis	Yes	No	No	No		No	Yes
Reason for initiating tocilizumab	Arthritis and ILD	*STAT3* GOF diagnosis	Severity of the disease and multi-organ involvement	Refractory hypoxemia	ILD	Worsened respiratory status and enteropathy	Severe symptoms of cardiac decompensation
Dose	240 mg monthly	12 mg/kg/15 days	12 mg/kg/4 months	Unknown dose	Unknown dose	400 mg weekly increased to 500 mg weekly	Refused
Improvement	Yes	Yes	Yes	Yes	No	No	-
Reason for initiating JAK inhibitor	-	-	Combination therapy	Combination therapy	Combination therapy	Worsened respiratory status, worsened portal hypertension, enteropathy, and malnutrition	-
JAK inhibitor dose	-	-	Ruxolitinib 15 mg BID	Ruxolitinib 2.2 mg/kg/day	Ruxolitinib 5 mg BID	Ruxolitinib 7.5 mg BID, then increased to 10 mg BID	-
Survival status	Alive	Alive	Alive	Alive	Alive	Dead	Dead
Cause of death	-	-	-	-	-	Respiratory failure complicated by pulmonary hemorrhage with aspergillosis, Torulopsis glabrata, and Pseudomonas sepsis	Progressive lung disease and heart failure

ILD, interstitial lung disease; FTT, failure to thrive; LAD, lymphadenopathy; LIP, lymphocytic interstitial pneumonitis; SPM, Splenomegaly; HTM, hepatomegaly; M, male; F, female.

## 5 Discussion

IEIs are primarily caused by single gene mutations. The International Union of Immunological Societies Expert Committee has identified 583 distinct genetic mutation-induced immunodeficiency disorders, with the latest classification updated in 2024.

In an international cohort study, Leiding et al. (7) collected information of 191 patients (88 from Europe, 71 from the United States, and 32 from the rest of the world) with reported or new germline *STAT3* GOF mutations. Patients with *STAT3* GOF mutations predominantly presented with lymphoproliferation, characterized by elevated frequencies of double-negative (CD4^−^ CD8^−^) T cells, autoimmune hematopenia, and hypogammaglobulinemia. Symptoms affecting the endocrine, digestive, and respiratory systems were common, whereas the involvement of the nervous and cardiovascular systems was generally less pronounced. Most patients presented with three or more disease phenotypes, and 43% of the patients had ILD, of which 26% also had bronchiectasis. Approximately 50% of the patients with ILD exhibited mutations in the DNA-binding domain at the genetic level. To date, no definitive evidence has been reported to supporting a genotype–phenotype correlation. However, in most cases, ILD progresses at a slow rate (8). Only a small number of patients experience infantile ILD, which is usually more aggressive (5, 9). STAT3 may be involved in pulmonary fibrosis through its role in fibroblast transformation, potentially contributing to the development of ILD in patients with *STAT3* GOF (10). Various cytokines activate STAT3. Specifically, IL-6 binds to its corresponding receptors on the cell membrane, triggering downstream JAK phosphorylation, which in turn leads to STAT3 phosphorylation and homodimer formation (11, 12). The homodimeric STAT3 then enters the nucleus through the nuclear membrane and mediates RNA transcription (9, 13, 14). Abnormally activated STAT3 signaling promotes TGF-β-induced fibroblast activation and collagen release at the molecular level, potentially contributing to the persistence of ILD in patients (15). Notably, hyperactive STAT3 may disrupt growth hormone (GH) biosynthesis by forming heterodimers with STAT5, thereby interfering with STAT5-mediated GH synthesis pathways and ultimately leading to GH deficiency (16). Although the patient in our case exhibited normal height (50 cm) and weight (3.9 kg) at birth, the FTT observed during follow-up likely resulted from the synergistic effects of chronic malnutrition and underlying disease progression. Our patient also exhibited classic hematological abnormalities, including cytopenia and hypogammaglobulinemia, accompanied by arthritis and ILD. Although double-negative (CD4^−^ CD8^−^) T cell quantification was not performed, the patient demonstrated disease-associated CD8^+^ T cell expansion alongside a reduction in CD4^+^ T cells, consistent with the typical immunophenotypic profile of this pathology (Table 1). The diagnostic delay prevented accurate assessment of the ILD onset timeline. Thoracic CT performed at our hospital demonstrated mediastinal displacement and bronchiectatic changes, highlighting the importance of early radiographic detection and prompt therapeutic initiation to mitigate disease progression.

Although clear and well-established treatment guidelines for this disease remain unavailable (17, 18), targeted therapies, such as tocilizumab and ruxolitinib, are preferred (19). JAK inhibitors or combination therapy could be more effective in controlling clinical symptoms (Table 2). However, one patient whose condition could not be controlled with tocilizumab alone died after switching to JAK inhibitors (Table 2). Owing to insufficient clinical data, the superiority of combination therapy remains unproven. JAK inhibitors are widely used to treat conditions associated with *STAT3* GOF mutations. Our decision to administer tocilizumab was based on its proven effectiveness in controlling arthritis and ILD, which are common comorbidities in rheumatic and autoimmune disorders. In addition, JAK inhibitors are associated with severe adverse effects in the pediatric population. It may increase susceptibility to infections and potentiate adverse outcomes in the cardiovascular, gastrointestinal, and neurological systems. Notably, the patient exhibited significant improvements for both arthritis and ILD following tocilizumab treatment. Additionally, hematopoietic stem cell transplantation (HSCT) has emerged as a significant treatment option for cases progressing to severe conditions such as severe immunodeficiency, refractory cytopenia, and high malignancy risk (20). A previous international study reported a survival rate of 61% among 18 patients with *STAT3* GOF mutations following HSCT, with infectious complications and graft-versus-host disease being the primary causes of mortality (21). The patient in our case remains clinically stable with tocilizumab therapy, and HSCT is not being considered at this time.

In conclusion, the clinical heterogeneity of the disease, coupled with limited current understanding, often results in misdiagnosis and underdiagnosis. When *STAT3* GOF is suspected, early WES should be prioritized. The timely administration of tocilizumab and JAK inhibitors is essential for patients with ILD. When disease control is inadequate, combining tocilizumab with JAK inhibitors may be considered as a preferred strategy. A deeper understanding of this disease will improve the detection rate, and we look forward to future therapeutic advances and more detailed mechanistic insights into this condition.

## Data Availability

The original contributions presented in the study are included in the article/Supplementary Material, further inquiries can be directed to the corresponding authors.
