# Supplementary figures and images for: Successful anti-IL-6 treatment for interstitial lung disease associated with STAT3 gain-of-function: a case report and literature review
Source: Front Pediatr. 2025 Jul 9;13:1577746. doi: 10.3389/fped.2025.1577746 (PMC12283785; doi:10.3389/fped.2025.1577746)

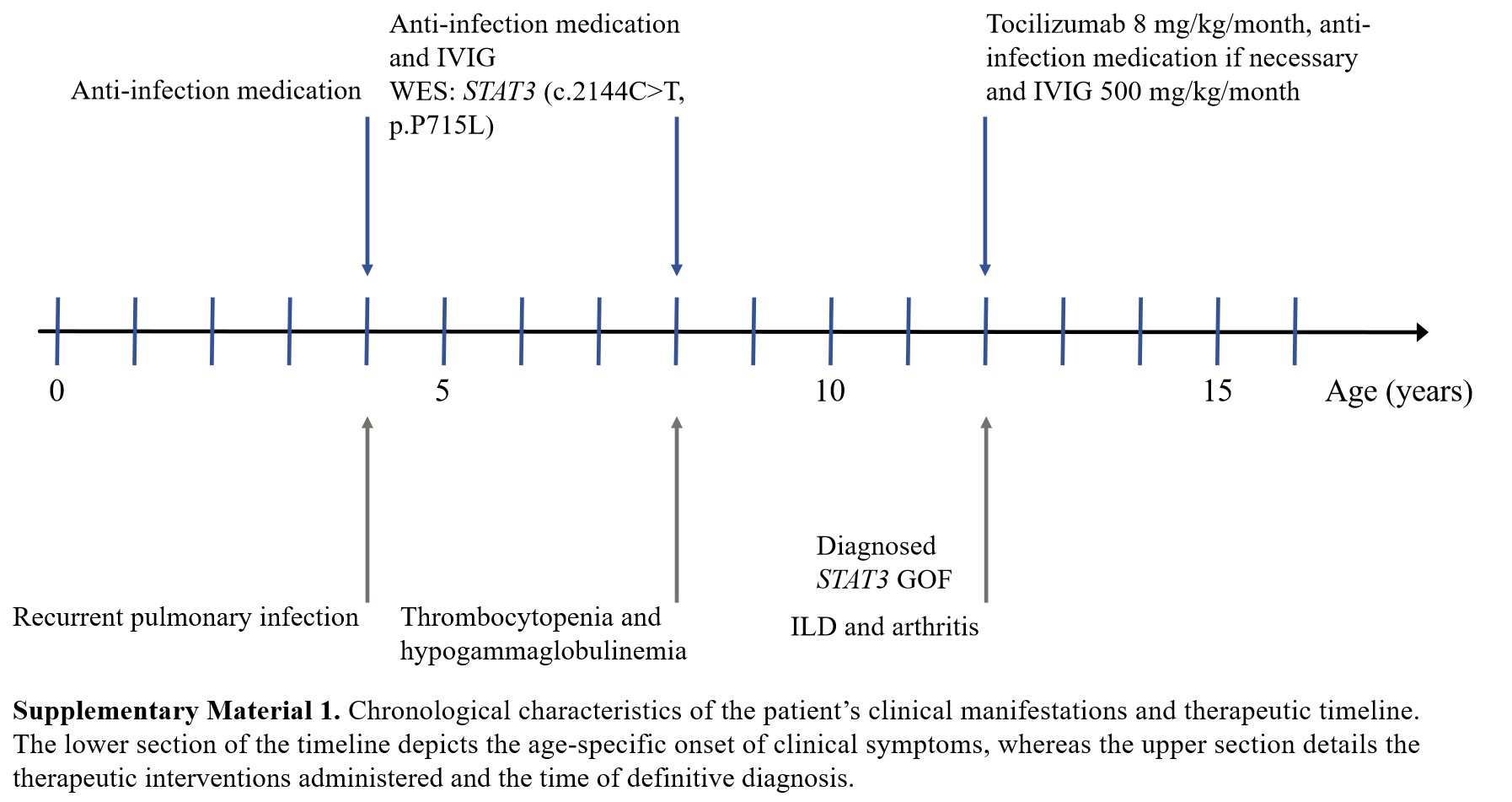

Supplement: Supplementary file 1 [file Image1.tif]
